# Neuroendocrine Tumors: Clinical, Histological and Immunohistochemical Perspectives and Case Report—Mature Teratoma in a 16-Year-Old Girl

**DOI:** 10.3390/pathophysiology28030025

**Published:** 2021-08-27

**Authors:** Elżbieta Sowińska-Przepiera, Dariusz Starzyński, Anhelli Syrenicz, Ireneusz Dziuba, Barbara Wiszniewska, Sylwia Rzeszotek

**Affiliations:** 1 Department of Endocrinology, Metabolic and Internal Diseases, Pomeranian Medical University (PMU), 71-252 Szczecin, Poland; elasowprzep@wp.pl (E.S.-P.); starzynskimd@gmail.com (D.S.); anhelli@asymed.ifg.pl (A.S.); 2 Faculty of Medicine, University of Technology, 40-555 Katowice, Poland; mmid@wp.pl; 3 Department of Histology and Embryology, Pomeranian Medical University (PMU), 70-111 Szczecin, Poland; barbara.wiszniewska@pum.edu.pl

**Keywords:** germinal neoplasm, neuroendocrine tumor, ovarian tumor, pediatric gynecology, teratoma, IHC

## Abstract

A mature teratoma is a germinal neoplasm that differentiates from embryonic multipotent cells into three germ layers. There may also be glandular tissue. The literature describes a total of 658 cases of ovarian neuroendocrine neoplasms, mainly in women over 40 years of age. The authors, together with a systemic review, present a case of a 16-year-old girl diagnosed with and treated for a neuroendocrine tumor. Case description: A 16-year-old girl visited the Paediatric Gynaecology Outpatient Clinic because of abdominal pains that intensified during menstruation. Standard painkillers and diastolic drugs were ineffective. An ultrasound examination revealed a large tumor with a heterogeneous structure in her right ovary. A sparing operation was carried out. During laparotomy, the lesion was enucleated, leaving healthy tissue. Histopathological examination revealed the typical features of teratoma, as well as the coexistence of a G1 neuroendocrine tumor. Immunohistochemical examination (IHC) showed the presence of markers characteristic for this type of tumor. The patient requires constant monitoring in the Endocrinology and Oncological Gynaecology Clinic. Conclusion: Tissue of neuroendocrine neoplasm within a teratoma is rare in this age group of patients; thus, there are currently no standards for long-term follow-up. This case adds to the body of evidence and demonstrates a possible good prognosis with non-aggressive behavior in G1 neuroendocrine tumors and teratomas in young patients.

## 1. Introduction

Primary germ cell (PGC) migration and their invasion of the genital ridges directs the development of the indifferent gonads into a testis or ovary, as well as differentiation of the germ cells. Aberrations during this process can lead to the appearance of germ cells anywhere, usually in the midline location, which is one of the steps in the PGC migration pathway from the yolk sac [1,2]. Ectopic germ cells can cause benign and malignant neoplasms—germ cell tumors (GCT) [3].

Teratomas are a common form of GCT. They can arise congenitally or can develop in childhood; in addition, especially with regard to gonadal teratomas, they develop over the course of life. However, most cases are seen in people in their reproductive years [4]. Mature cystic teratoma (MCT) occurs in 10–20% of all ovarian cancers, and the average age of occurrence is perimenopausal [5,6]. MCT in pure form is benign; however, it may also undergo a malignant change in any of its somatic elements [7].

The tissue of mature teratomas may be glandular, the most common being that of the thyroid gland, the so-called ovarian goiter, which occurs in approximately 3–5% of teratomas [8].

Neuroendocrine tumors (NETs) are considered rare in the general population, with an estimated incidence of 4–8 cases per 100,000 people, both in clinical observations and in post-mortem studies [9]. NETs are a heterogeneous group of neoplasms that arise from cells of the endocrine and nervous systems and can be functional or non-functional [10,11,12]. In recent decades, the incidence of these rare tumors has increased significantly, and their malignant potential (NETs were previously considered relatively benign) has been reported [13,14]. However, this upward trend may be due to improved diagnostics and a standardized classification system [15,16].

Ovary neuroendocrine (tumors) neoplasms (oNETs) account for approximately 0.3% of gonadal tumors [17]. oNETs differ morphologically, including poorly differentiated variants and well-differentiated low-grade variants called carcinoids [18]. Dispersed neuroendocrine cells can give rise to neuroendocrine tumors in all tissue types also in combination with teratoma. Primary ovarian carcinoid tumors are extremely rare, usually appear in perimenopausal women and their behavior is poorly predictable.

In around 75% of cases, oNETs occur unilaterally as a component of a mature teratoma. In approximately 15% of cases, they occur bilaterally in the form of a cyst, mucinous or Brenner tumor [19].

In 25–30% of cases, NETs may be associated with carcinoid syndrome, which means that they release biologically active substances into the blood. The symptoms of carcinoid syndrome depend mainly on the substance released from the cancer cells into the blood. [20]. The paroxysmal nature of the symptoms is characteristic, and they include face and neck redness, tachycardia, dizziness, excessive sweating, diarrhea, watery stools with accompanying abdominal pain, weight loss and symptoms of bronchospasm, such as dyspnea and wheezing. Carcinoid syndrome is especially common in tumors of the ileum and jejunum, but it can also occur in NETs of the bronchi, ovaries and other tumor locations. In the gut, the symptoms of carcinoid syndrome are usually seen in people with advanced disease who have metastatic foci in the liver; however, in the bronchial localization, they may occur earlier since the peptides secreted by the tumor are not degraded in the liver [21,22].

In this case, NET was present as a histological component of a teratoma and was hormonally inactive in a 16-year-old patient.

## 2. Case Presentation

Written and signed informed consent was obtained from the patient. A 16-year-old female patient came to the clinic due to intensified pain during menstruation. She used painkillers, mainly paracetamol, which were ineffective. The patient had been menstruating for 3 years. She did not report chronic diseases or prior surgeries. The physical examination showed normal somatic gender development (according to Tanner Th4P4A4 scale) adequate for her age.

**Figure 1:**
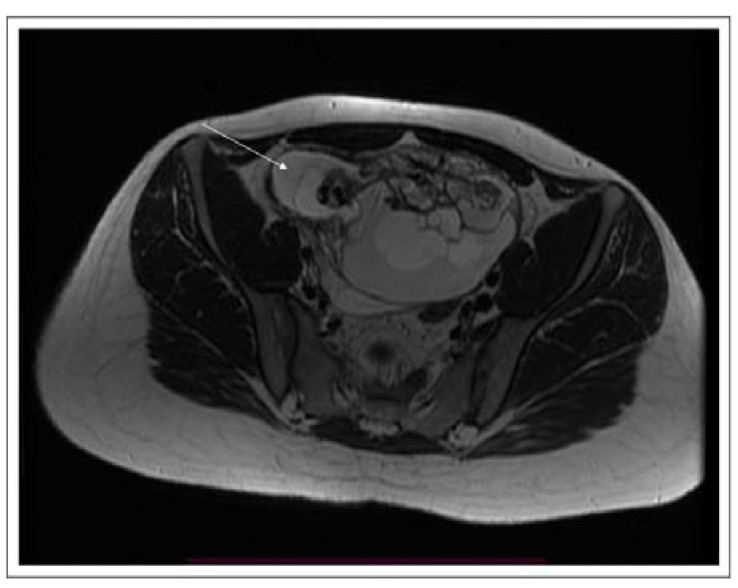
NMR of the pelvis minor in the transverse plane. The arrow indicates the tumor in the right ovary.

Routine ultrasound examination revealed the presence of a large, heterogeneous tumor (14.0 × 10.0 × 10.5 cm) in the right ovary. Standard laboratory tests showed no abnormalities. The concentrations of germinal (α-fetoprotein, AFP; human chorionic gonadotropin, hCG; lactate dehydrogenase, LDH) and epithelial (CA-125 tumor antigen, CA-125; carcinoembryonic antigen, CEA) tumor markers were within the normal ranges. Computed tomography (CT) and subsequent nuclear magnetic resonance (NMR) revealed a large (15 × 13 × 9 cm), non-homogeneous, encapsulated tumor in the projection of the right ovary. There were no signs of infiltration or enlargement of the lymph nodes. The remaining genital structures, including the left ovary, were unaffected (Figure 1).

Due to the patient’s young age, a sparing laparotomy was performed. The tumor of the right ovary, a periapical cyst and 5 mL of fluid from the abdominal cavity were removed. No postoperative complications occurred.

Histopathological examinations reveled a mature cystic teratoma with a G1 neuroendocrine neoplasm focus; immunohistochemical (IHC) analysis revealed approximately 1% Ki-67 and synaptophysin-positive (Syp) areas, while chromogranin A (CgA) was weak with a “dot-like” cytoplasmic pattern. Additionally, cytokeratin 19 (CK19), neural cell adhesion molecule (CD56) and neuron-specific enolase (NSE) were positive in many tests (please check Supplementary Materials). In the abdominal cavity, no tumor cells were found.

Due to the presence of tissue with NET features, the patient’s case was presented to a team of experts to determine further management and monitoring of oncological treatment.

It was recommended to monitor the patient for the development of carcinoid syndrome and to determine the concentration of NET tumor activity markers: chromogranin A and 5-hydroxyindole acetic acid (5-HIAA).

The patient’s clinical symptoms as well as biochemical and imaging results are presented in Table 1.

An elevated 5-HIAA concentration was found (18.85 mg/24 h; normal: 2–6 mg/24 h), which may suggest the presence of local foci or cancer metastases. Preoperative diagnostics did not indicate a hormonally active neuroendocrine tumor due to the lack of typical clinical symptoms of carcinoid syndrome, so preoperative 5HIAA determination in 24 h urine collection was not performed. On the other hand, postoperative 5HIAA determination in 24 h urine collection suggests incomplete removal of the ovarian tumor from NET, NET foci with a different location or metastatic changes. However, imaging diagnostics using MR, PET/CT with Ga68 and receptor scintigraphy did not reveal changes confirming this suspicion, which requires further observation and control. The concentration was measured according to the current rules in order to exclude false-positive results [20]. Chromogranin A was within the normal range (28.21 ug/L; normal: 0–100 ug/L).

**Figure 2:**
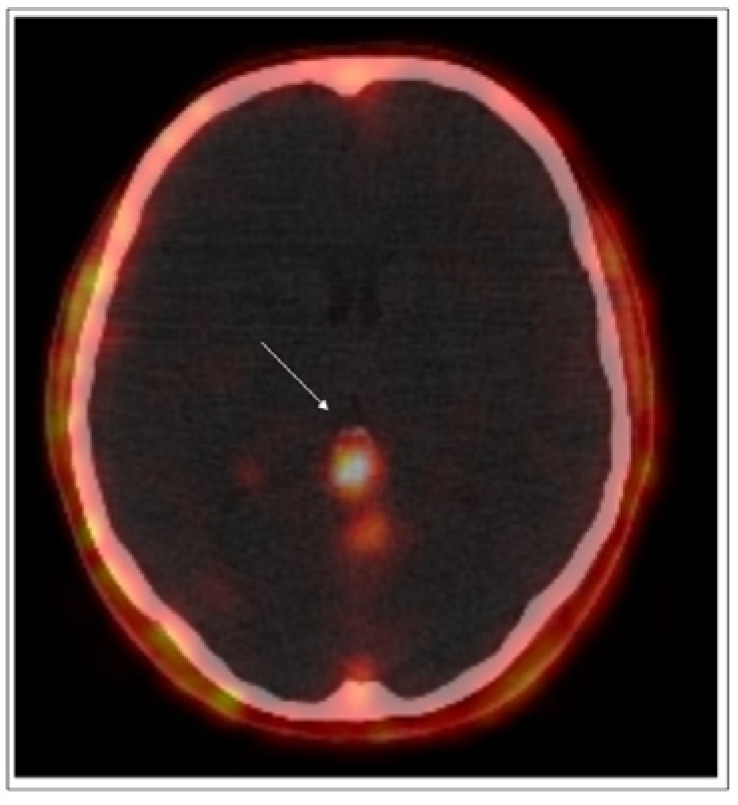
PET/CT test (68Ga-DOTA-peptide). The arrow indicates the increased somatostatin receptor expression in the pineal gland.

CT scans were performed to compare the pre- and post-surgery conditions, followed by technetium (Tc)-99m receptor scintigraphy, which did not detect pathological foci showing increased expression of the somatostatin receptor. The positron emission tomography/computed tomography (PET/CT) examination with Gallium-68 DOTATATE (68 Ga-DOTATATE) radiotracer revealed a small focal point with increased somatostatin receptor expression in pineal topography. NMR imaging was recommended, which showed a cystic structure in this area (Figure 2).

Pelvic sonography and NMR showed no additional tumor foci or metastases. The oncological diagnostics have been completed at this stage. The patient is currently under constant supervision.

## 3. Methods

### IHC

The dissected tumor was fixed with 10% formalin for at least 24 h and then washed with absolute ethanol, absolute ethanol with xylene (1:1) and xylene. Then, after the tissues were saturated with liquid paraffin, the samples were embedded in paraffin blocks. Using a microtome (Microm HM340E, Walldorf, Germany), serial 3–5 µm sections were taken and placed on poly-l-lysine-coated microscope slides (Thermo Scientific, Leicestershire, UK; Cat. No. J2800AMNZ). The sections were deparaffinized in xylene and rehydrated in decreasing ethanol concentrations before being used for H&E and IHC staining. To reveal epitopes for IHC, the sections were boiled twice in Target Retrieval Solution (DakoCytomation, Glostrup, Denmark; S2367, S2369) in a microwave oven (700 W, twice for 5 min). After cooling and washing with PBS, endogenous peroxidase was blocked with a 3% perhydrol solution in methanol; then, slides were incubated overnight at 4 °C with primary antibodies against: Ki-67 (Dako Agilent, Santa Clara, CA, USA; GA626, ready to use), synaptophysin (Syp, Dako Agilent, Santa Clara, CA, USA; GA660, ready to use), cytokeratin CK19 (CK19, Dako Agilent, Santa Clara, CA, USA; GA615 ready to use), neural cell adhesion molecule (CD56, Dako Agilent, Santa Clara, CA, USA; M7304, final concentration 1:200), CD34 molecule (CD34, Dako Agilent, Santa Clara, CA, USA; GA632, ready to use), platelet endothelial cell adhesion molecule (CD31, Dako Agilent, Santa Clara, CA, USA; GA610, ready to use), podoplanin D2-40 (D2-40, Dako Agilent, Santa Clara, CA, USA; M3619, final concentration 1:200), thyroid transcription factor 1 (TTF-1, Dako Agilent, Santa Clara, CA, USA; M3575, final concentration 1:200), (CK20, Dako Agilent, Santa Clara, CA, USA; M7019, final concentration 1:200), homeobox protein CDX-2 (CDX2, Dako Agilent, Santa Clara, CA, USA; M3636, final concentration 1:200), thyroglobulin (Tg, Dako Agilent, Santa Clara, CA, USA; M0781, final concentration 1:200), calcitonin (CT, Dako Agilent, Santa Clara, CA, USA; A0576, final concentration 1:200), chromogranin A (CgA, Dako Agilent, Santa Clara, CA, USA; M0869, final concentration 1:200), neuron-specific enolase (NSE, Dako Agilent, Santa Clara, CA, USA; M0873, final concentration 1:200), glial fibrillary acidic protein (GFAP, Dako Agilent, Santa Clara, CA, USA; Z0334, final concentration 1:200).

Antibodies were diluted in Antibody Diluent if required (Dako Agilent, Santa Clara, CA, USA; S3022). To visualize the antigen–antibody complex, a Dako LSAB+System-HRP was used (DakoCytomation, Glostrup, Denmark; K0679), based on the reaction of avidin–biotin–horseradish peroxidase with DAB as a chromogen, according to the included staining procedure instructions. Sections were washed in distilled H_2_O and counterstained with hematoxylin. For a negative control, specimens were processed in the absence of a primary antibody. Positive staining was determined microscopically (Leica DM5000B, Wetzlar, Germany) by visual identification of brown pigmentation.

## 4. Results

### 4.1. Histopathological Findings

The tumor mass, with a size of 13 × 10 × 8 cm, on a multicystic cross-section, was loose, due to the predominance of fat and sebum (tallow), and filled with hairs, teeth and mucogelatinous content.

#### 4.1.1. TERATOMA

**Figure 3:**
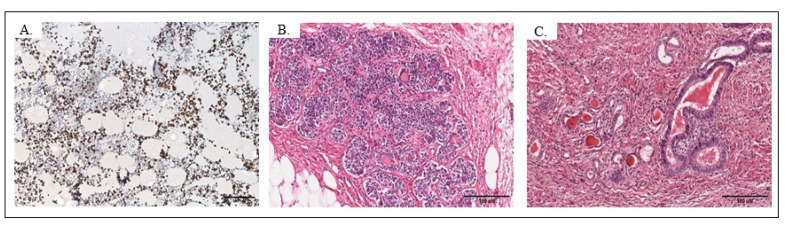
HE staining of the strumal carcinoid of the ovary, objective magn. ×20, Leica DM5000B, Wetzlar, Germany. Ki-67 expression was limited to the bone marrow area of the teratoma (**A**); rounded or arched cords of columnar or pyramidal cells typical for the glomerulosa zone of the adrenal cortex were visible within the strumal carcinoid (**B**); thyrocytes with variability in shape—cuboidal, flattened or even slightly columnar—were observed surrounding the colloid (**C**).

Microscopic examination of the ovary tumor revealed tissues derived from mesenchyme, mesoderm, endoderm, ectoderm and neuroectoderm. Mature hyaline cartilage encapsuled partially with connective tissue, occasionally with bone trabeculae, and some components of the epiphyseal plate were identified. Scattered islands of mucous and serous glands were present, especially in the area of epithelium typical for the bronchi. The cells of the bone marrow were the only population within this tumor showing the expression of Ki-67 (Figure 3A). Irregular lymphoid nodules and the accumulation of lymphocytes were present in the connective tissue of the mucosa of the alimentary tract. The mucosa was lined with different types of epithelia. Most of the secretory gland-like structures were not accompanied by ducts. The most abundant tissue type in this ovarian teratoma was skin with pilosebaceous units. There was the coexistence of rounded or arched cords of columnar or pyramidal cells typical for the glomerulosa zone of the adrenal cortex (Figure 3B) and thyroid gland. Thyrocytes with a tendency to form follicular structures showed variability in shape—cuboidal, flattened or even slightly columnar—and were located around the colloid (Figure 3C). They showed no malignant features similar to pleomorphic and anaplastic cells. However, in some cells, cytoplasm was scanty and pale with large nuclei with dense chromatin and invisible nucleoli, and, in the others, the nucleus was typically vesicular with visible nucleoli. Signet ring cells were invisible. There was no evidence of proliferation, solid growth of the tumor cells or necrosis. Glandular structures lined with cuboidal or columnar epithelial cells with bright cytoplasm and flattened nuclei were noticeable. Structures were separated by a delicate fibrovascular tissue.

#### 4.1.2. NET

Within the teratoma, small, round cells with coarse, salt and pepper nuclear chromatin were observed. Morphological findings indicated that a carcinoid tumor was present in the wall of the mature cystic teratoma. The architecture of the NET was rather glandular. Tumor cells were arranged in acini and trabeculae, having round to oval nuclei, stippled chromatin and abundant granular eosinophilic cytoplasm. Other elements of this teratoma with co-occurrence of a G1 neuroendocrine tumor resembled a heavily disturbed tissue type without sufficient distinguishable characteristic features.

### 4.2. Immunohistochemical Findings of NET

**Figure 4:**
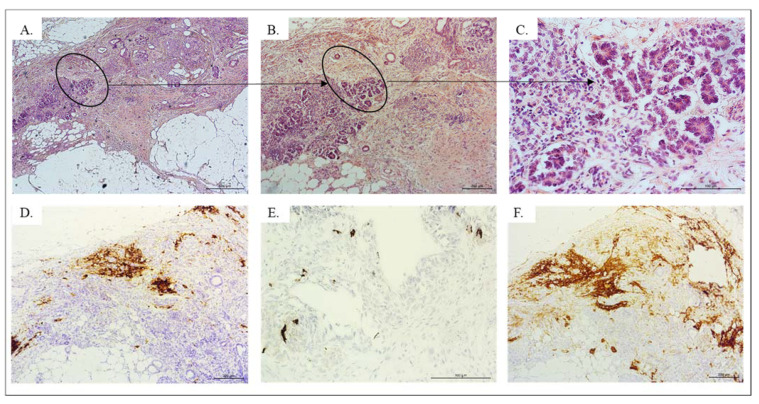
HE and IHC staining of the NET, Leica DM5000B, Wetzlar, Germany. Localization of the NET within the teratoma: ((**A**). objective magn. ×5; (**B**). objective magn. ×20; (**C**). objective magn. ×40); Syp in cells of the NET ((**D**). objective magn. ×20); CgA was weak, with “dot-like” cytoplasmic pattern ((**E**). objective magn. ×40), CD56 positive area ((**F**). objective magn. ×20).

Syp was expressed in the cells of the NET (Figure 4). Cells of the tumor were negative for CK20, CDX2 and Tg. A positive signal was observed only in some cells of the carcinoid for CK19 and CD56. The areas associated with NET included blood vessels and lymphatic vessels, which were evaluated with CD34, CD31 and podoplanin D2-40. Within the area of thyroid tissue, a negative signal for CT and Tg and positive for TTF1 was identified, accompanied by a strong positive reaction for markers of the endothelium of the blood and lymphatic vessels.

### 4.3. Immunohistochemical Findings of Teratoma

**Figure 5:**
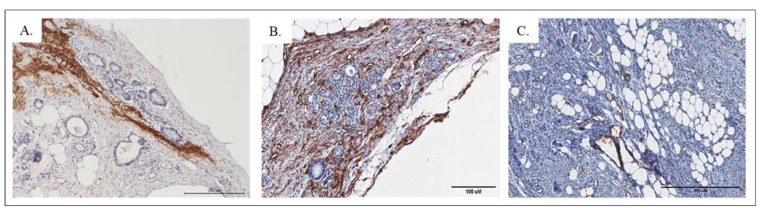
Area of carcinoid with GFAP ((**A**). objective magn. ×20), CD 34 ((**B**). objective magn. ×10) and CD31 ((**C**). objective magn. ×10).

Vascular proliferations identified with high expression of CD34 (Figure 5B) and CD31 (Figure 5C) positive cells were observed within the strumal area. Bundles of the nerve fibers were common. Occasionally, areas with neural differentiation positive for GFAP (Figure 5A) and reaching blood vessels were visible.

## 5. Discussion

### NET

The teratoma diagnosed in this patient with NET tissue is extremely rare in this age group (Table 2); thus, there are no standards for long-term follow-up. Preoperative differentiation of benign and malignant changes in the ovary in young premenopausal women is difficult because no test or algorithm is clear about the accuracy and differentiation of these changes. The exceptions are embryonic tumors with elevated levels of specific tumor markers such as α-FP or hCG. In addition, approximately 10% of ovarian lesions are suspected to be metastatic and not of ovarian origin [23]. The diagnosis of an ovarian proliferative lesion (hyperplasia) exceeding 6 cm in premenopausal women is an indication for diagnostics and surgical treatment as the possibility of a malignant process should be considered [24]. In young women before their peri-reproductive period, as in our patient, if technically possible, surgery should be performed using the least invasive technique, i.e., laparoscopy. However, after a comprehensive surgical evaluation, the surgical technique should be carefully chosen. The preoperative assessment of malignant potential is necessary to plan the optimal surgical strategy [25,26].

Primary and secondary ovarian NETs have similar histological growth mechanisms but differ in clinical course, treatment and prognosis [27].

A previous analysis of 329 oNET cases showed that if these tumors are not a component of teratomas, they tend to be larger in size and have more frequent general and liver metastases and a lower 5-year survival rate [19].

Ki-67 is an independent prognostic biomarker for patients. The Ki-67 value is determined by immunohistochemical methods with mindbomb homolog 1 (MIB1) antibody; the number of mitotic figures is also evaluated. According to the current WHO 2019 [28] classification, neuroendocrine neoplasms (NENs) are divided into highly differentiated NET G1 with Ki-76 < 3%, NET G2 with Ki-67 of 3–20%, NET G3 with Ki-67 > 20%; low differentiated neuroendocrine carcinomas (NECs) with Ki-67 > 20%; microcellular (SCNEC) or giant cell (LCNEC); and mixed neuroendocrine neoplasms (MiNEN) with a variable Ki-67 proliferative index. In this case, this proliferation indicator was identified only within the area of the bone marrow and did not align with the high number of CD31- and CD34-positive cells. It is worth noting as the stage of vascular maturity can be an important differentiating factor in teratoma grading criteria [29]. In predictions, attention should be paid to vascular invasion and infiltration of the capsule [30].

CT produced primarily by the parafollicular cells (C-cells) can also be an IHC marker for the metastatic medullary thyroid carcinoma. In this case, we did not find a positive signal for CT. The relationship between functionally inactive (negative for Tg, CT and intermediate expression of TTF1) thyroid tissue and a neuroendocrine marker-secreting tumor present within the teratoma can be connected to the theory of the stem cell origin of this kind of tumor. The clinically silent thyroid tissue observed within this teratoma could be connected to improper migration, aberrant differentiation or, as some authors report, transport of the thyroid cells through the lymphatic vessels. It was challenging to clearly differentiate some features: follicles were heterogeneous in shape and a tissue pattern was unclear, with pleomorphic nuclei and, in some areas, crowded nuclei. However, cellular proliferation was invisible and the majority of the tissue had cells that were regular in shape [31].

In this case, expression of CK19 was observed in the lining of the alimentary tract but there was no evidence of cellular proliferation in this area. This is important as, in some carcinomas, high co-expression of CK19 and Ki-67 is an undesirable prognostic marker [32].

In this case, the focus of the highly mature neuroendocrine carcinoid was identified with a Syp, weak CgA, negative CK20 and positive CD56. Synaptophysin as a specific marker for neuroendocrine tumors showed cytoplasmic authoritative results. The combined use of CK19 and CD56 is helpful in discriminating papillary thyroid carcinoma and its variants from other mimicking thyroid lesions [33]. We did not find co-expression of these markers in the area of the thyroid tissue.

Podoplanin is a protein expressed in a variety of cell types, but the positive reaction with podoplanin D2-40 antibody is a known marker of the lymphatic vessels and their development [34]. In the studied case, D2-40 was visible as typical membranous, strong and diffuse staining in lymphatic vessels, but it was also present at the periphery of the sebaceous glands and in the deeper parts of the stratified squamous epithelium, limiting the tumor. The role of podoplanin in tumor invasion is under discussion; however, it has been observed that podoplanin is upregulated in the outer edge of the tumor mass and, in some cases, is associated with poor prognosis [35].

Our patient had a G1-type oNET in combination with dermoid, without carcinoid syndrome, which appears to be prognostically favorable. In long-term observation, limited and mainly casuistic data concerning the proliferation index in the prediction of oNETs have been reported [36].

According to the recommendations of the Polish Network of Neuroendocrine Tumors, surgery is the main method for the treatment of oNET. In the case of G1-type tumors, a joint ovariectomy–tumor excision is recommended, with the excision of the uterus with adnexa and a larger network for malignant tumors [36].

The European Society of Medical Oncology (ESMO) guidelines for non-epithelial neoplasms advise that young patients undergo sparing treatment to preserve fertility. Fertility-saving surgeries seem to be safe, with excellent survival after long-term follow-up, such as the inclusion of more aggressive treatments. Patients with stage IA G1 teratomas do not require neoadjuvant chemotherapy. Chemotherapy in more advanced stages (IA G2-G3, IB-IC) is controversial. Patients who have abandoned chemotherapy should be actively monitored. In the case of oNET teratoma, specialist imaging tests are recommended, e.g., PET/CT, CT, receptor scintigraphy, MRI and assessment of the concentrations of markers specific for these types of tumors [37]. oNETs co-occurring with teratomas are characterized by good prognosis, rare metastases and symptoms of carcinoid syndrome; however, due to the lack of long-term observations of these rare cases, the National Comprehensive Cancer Network guidelines do not cover oNETs (www.nccn.org, version 2.2021, accessed on 24 August 2021).

**Table 2:** Age of patients described in case reports of carcinoids.

Year	Author	Diagnosis	Age
2020	Maccora et al. [38]	mature teratoma with insular carcinoid tumor of the ovary	68
2019	Chai et al. [39]	strumal carcinoid tumor of the ovary	63
2019	Yan et al. [40]	multiple endocrine neoplasia type 1-related atypical ovarian carcinoid	30
2019	Borghese et al. [41]	bilateral MCT with foci of ovarian strumal carcinoid, developed lymph node para aortic metastasis after 30 years from primary diagnosis	?
2019	Hsu et al. [42]	primary ovarian mucinous carcinoid tumor, atypical type, very aggressive	33
2018	Ishida et al. [43]	stromal carcinoid of the ovary	68
2018	Macháleková et al. [44]	stromal carcinoid of the ovary	46, 52
2018	Niu et al. [45]	carcinoid arising from the teratomatous bronchial mucosa in an ovarian MCT	22
2017	Fiore et al. [46]	goblet-cell carcinoid of the ovary	18
2016	Kim [47]	carcinoid tumor of the trabecular type arising from an MCT in ovary	25
2016	Erdenebaatar et al. [48]	insular carcinoid tumor of the ovary with a trabecular component	70
2016	Kim et al. [49]	primary ovarian mixed strumal and mucinous carcinoid arising in an ovarian MCT	39
2016	Vora et al. [17]	well-differentiated carcinoid tumor with no surface epithelial involvement, and a mature teratoma in the contralateral ovary, a mature teratoma; strumal carcinoid within the ovarian parenchyma; poorly differentiated carcinoma with neuroendocrine differentiation	40, 26, 63, 32
2015	Kim et al. [50]	primary ovarian carcinoid tumor with loss of neuroendocrine growth pattern, increased mitotic activity and large areas of coagulative tumor necrosis, atypical carcinoid	21
2015	Mieczkowska et al. [51]	primary ovarian carcinoid in mature teratoma of one ovary, co-existing with primary epithelial carcinoma of another ovary	
2015	Târcoveanu et al. [52]	ovarian strumal carcinoid and cystic lymphangioma	55
2015	Muller et al. [53]	ovarian strumal carcinoid (peptide YY producing)	34
2014	Goldman et al. [54]	secondary to carcinoid heart disease caused by a primary ovarian carcinoid tumor	61
2014	Gupta et al. [55]	primary ovarian carcinoid tumor simulating virilizing tumor of the ovary	62
2014	Kumar et al. [56]	carcinoid of the ovary	53
2013	Sulaiman et al. [57]	strumal carcinoid tumor stage 1A of the ovary	30
2012	Takatori et al. [58]	strumal carcinoid of the ovary (peptide YY producing)	48
2013	Hayashi et al. [59]	primary strumal carcinoid tumor of the ovary	45
2011	Takeuchi et al. [60]	strumal carcinoid tumor of the ovary primary strumal carcinoid tumor of the ovary	72 77
2011	Matsunami et al. [61]	strumal carcinoid tumor of the ovary (peptide YY producing)	45
2010	Marcy et al. [62]	lethal, malignant, metastatic struma ovarii	45
2010	Bai et al. [63]	primary ovarian trabecular carcinoid tumor	55
2010	Kurabayashi et al. [64]	primary strumal carcinoid tumor of the ovary with multiple bone and breast metastases	34
2009	Suneja et al. [65]	primary malignant melanoma in cystic teratoma of ovary	50
2009	Guney et al. [66]	primary carcinoid tumor arising in a mature cystic teratoma	54
2009	Gungor et al. [67]	primary ovarian carcinoid arising from a mature cystic teratoma	47
2008	Lagoudianakis et al. [68]	primary ovarian insular carcinoid tumor	44
2008	Gorin & Sastre-Garau [69]	strumal carcinoid tumor of the ovary	63
2007	Somak et al. [70]	primary carcinoid tumor of the ovary	55
2007	Morken et al. [71]	primary ovarian carcinoid tumor	70
2006	Chatzipantelis et al. [72]	insular carcinoid and mucinous cystadenoma of low malignant potential, arising in a cystic teratoma	57
2006	Karavolos et al. [73]	primary mucinous carcinoid tumor of the ovary	34
2005	Kopf et al. [74]	primary carcinoid tumor of the ovary	79
2003	Kuscu et al. [75]	ovarian carcinoid stage IA	47
2002	Matsuda et al. [76]	strumal carcinoid tumor of the ovary (peptide YY producing)	50
2000	McMurray [77]	benign left ovarian cystic teratoma, and a right carcinoid tumor of the ovary	57
1996	Kasantikul et al. [78]	primary ovarian carcinoid (insular, trabecular and mucinous components)	53
1996	Chou et al. [79]	primary ovarian carcinoid tumor	25
1995	Takemori et al. [80]	ovarian strumal carcinoid in association with dermoid cyst and mucinous cystadenoma in the same ovary	54
1995	Yaegashi et al. [81]	primary trabecular carcinoid of the ovary	43
1993	Kataoka et al. [82]	trabecular carcinoid tumor associated intimately with thyroid follicle-like structures, strumal carcinoid arising in a benign cystic teratoma	41
1992	Erhan et al. [83]	primary carcinoid tumor	55

## 6. Conclusions

The described pathology, extremely rare in this age group, was diagnosed by histopathological examination. Due to the young age of the patient and the need to preserve fertility, sparing surgery was applied. Using Tg as the only marker of thyroid tissue can create challenges. Long-term observation of these casuistic reports will allow for possible verification of the applied treatment. Nowadays, with the opportunity to evaluate and personalize the treatment of the patient, it is crucial to identify markers and pay attention to malignant transformations of initially not disturbing changes. It is encouraging that there is an increasing number of better described IHC markers that allow for diagnosis and the estimation of risk and prognosis. Unfortunately, NETs in the ovary are rare, so many predictive markers are based on observations of NETs present in the gastrointestinal tract. The stage of vascular proliferation and maturity is an important factor in tumor development and progression and should be carefully evaluated. This case adds to the body of evidence and demonstrates a possible good prognosis with non-aggressive behavior.

## Figures and Tables

**Table 1:** Clinical symptoms and results of consecutive biochemical and imaging diagnostic steps performed after operation in a 16-year-old girl with an ovarian neuroendocrine tumor after operation.

Symptoms of Carcinoid Syndrome	Absent	
Tumor marker concentrations	Chromogranin A	28.21 ug/L (normal: 0–100 ug/L)
	** 5-HIAA—mg/24 h	18.85 mg/24 h (normal: 2–6 mg/24 h)
Pelvic computed tomography (CT)	A 2.0 × 3.5 cm mass adjacent to the posterior wall of the uterus, slightly strengthening after the administration of contrast (postoperative lesions and the left part of the ovary)	
Tc99 receptor scintigraphy	No somatostatin receptor expression	
* PET/CT GAL68	Lack of somatostatin receptor expression in the operated area. A small focal point with increased receptor expression in pineal topography	
Magnetic resonance imaging (MRI)	Pineal topography showed an oval structure measuring 11 × 10 × 9 mm with features of a cyst	
Pelvic ultrasonography (USG) of the pelvis minor	Vague picture in the right appendage projection	
MRI of the pelvis minor	No lesions suspected of being cancerous were found	

* PET/CT GAL68—positron emission tomography with somatostatin analogue labeled with gallium isotope (68Ga-DOTA-peptide); ** 5-HIAA—5-hydroxyindoleacetic acid.

## Data Availability

The data presented in this study are available on request from the corresponding author. The data are not publicly available due to Patient’s privacy.

## Supplementary Materials

The following are available online at https://www.mdpi.com/article/10.3390/pathophysiology28030025/s1, Figure S1: IHC staining of the NET within the teratoma, Leica DM5000B, Wetzlar, Germany. Top row: negative signal for: A. CT ×20, B. Tg ×20, C. CDX-2 ×20, D. CK-20 x40. Bottom row: positive signal for: E. CK-19 ×20, F. TTF-1 ×20, G. podoplaninD2-40 ×40 H. NSE ×20.
