# Supplementary figures and images for: Neuroendocrine Tumors: Clinical, Histological and Immunohistochemical Perspectives and Case Report—Mature Teratoma in a 16-Year-Old Girl
Source: Pathophysiology. 2021 Aug 27;28(3):373–86. doi: 10.3390/pathophysiology28030025 (PMC8830444; doi:10.3390/pathophysiology28030025)

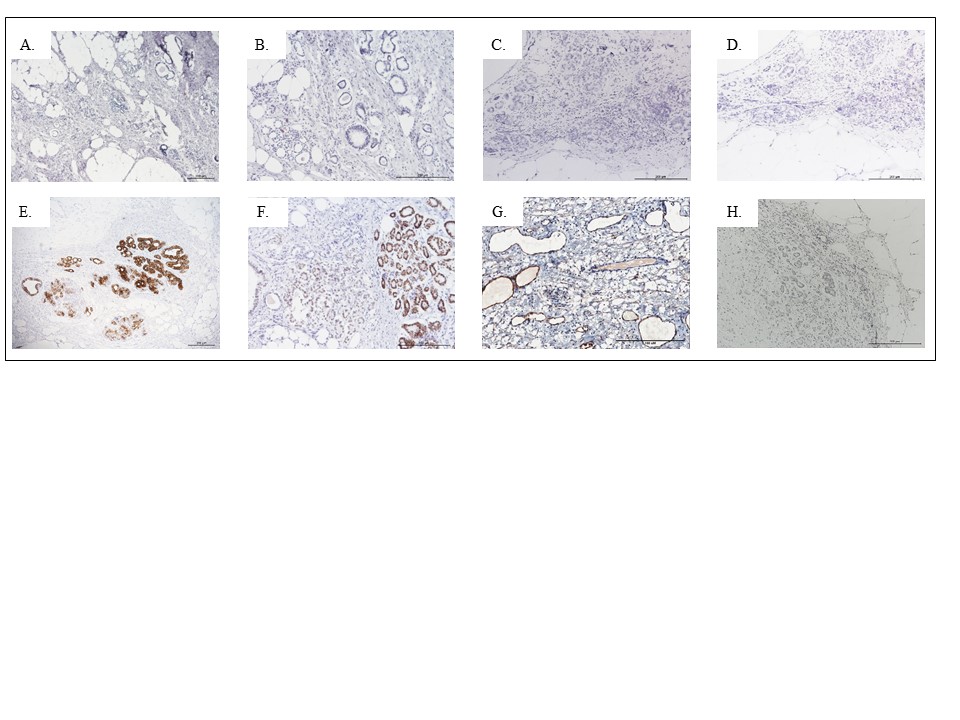

Supplement: Supplementary file 1 [file pathophysiology-28-00025-s001.zip › Figure S1.JPG]
